# *Malassezia* [mal″ə-sē′zhə]

**DOI:** 10.3201/eid3110.221743

**Published:** 2025-10

**Authors:** Fábio P. Sellera, Rüdiger D. Ollhoff, Fabio C. Pogliani

**Affiliations:** Universidade Metropolitana de Santos, Santos, Brazil (F.P. Sellera); Universidade de São Paulo, São Paulo, Brazil (F.P. Sellera, F.C. Pogliani); Pontifícia Universidade Católica do Paraná, Curitiba, Brazil (R.D. Ollhoff)

**Keywords:** Fungi, pityriasis versicolor, Pityrosporum, Louis-Charles Malassez, Carl Ferdinand Eichstedt, pathogenic fungus

**Figure 1:**
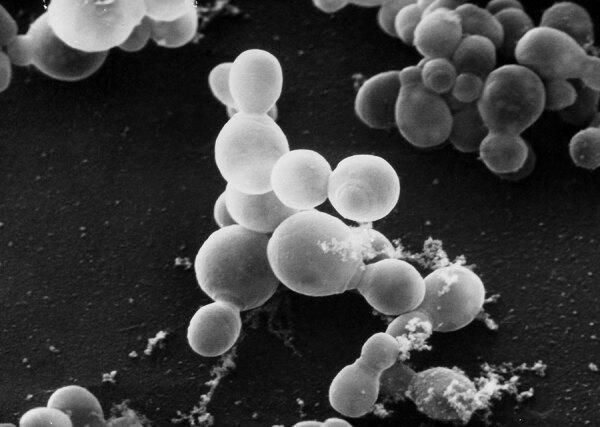
Scanning electron microscopic image of *Malassezia furfur* reproductive fungal spores. Source: Public Health Image Library, Centers for Disease Control and Prevention (https://phil.cdc.gov). Photograph credit: Janice Haney Carr.

*Malassezia* is a genus of basidiomycetous yeasts (Figure 1) that includes 18 validly published species, most of which are strictly lipophilic. These organisms are considered part of the normal cutaneous microbiota in humans and many animals. *Malassezia* spp. have also been associated with many skin diseases, including pityriasis versicolor, folliculitis, seborrheic dermatitis, dandruff, atopic dermatitis, and psoriasis.

**Figure 2:**
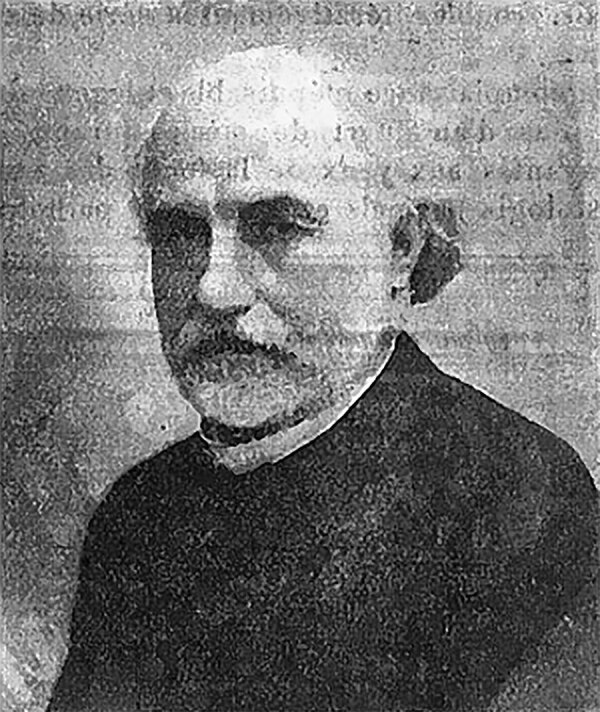
Louis-Charles Malassez (1842–1909), after whom *Malassezia* spp. fungi are named. Image reproduced under open license. Source: Paris City University Image and Portrait Bank (https://numerabilis.u-paris.fr/medica/banque-images/index.php).

In 1874, French histologist and anatomist Louis-Charles Malassez (Figure 2) isolated yeast cells from human dandruff scales and observed yeast with spherical and oval forms. Decades earlier, in 1846, Carl Ferdinand Eichstedt was the first to recognize the infectious nature of this fungus. He observed a journeyman with extensive pityriasis, which became subsequently known as Eichstedt’s disease.

The naming of this fungus was controversial because of disagreements over its classification, and it was initially named under the genus *Pityrosporum*. After years of debate, the genus was renamed *Malassezia* in 1984, honoring Malassez, who made major contributions to understanding this group of fungi, particularly in their taxonomic classification and lipophilic nature.
